# Voxel-Wise Adversarial FiboNet for 3D Cerebrovascular Segmentation on Magnetic Resonance Angiography Images

**DOI:** 10.3389/fnins.2021.756536

**Published:** 2021-11-16

**Authors:** Bin Guo, Fugen Zhou, Bo Liu, Xiangzhi Bai

**Affiliations:** Image Processing Center, School of Astronautics, Beihang University, Beijing, China

**Keywords:** index terms-class imbalance, image noise, CNN, GAN, medical image segmentation

## Abstract

Cerebrovascular segmentation is important in various clinical applications, such as surgical planning and computer-aided diagnosis. In order to achieve high segmentation performance, three challenging problems should be taken into consideration: (1) large variations in vascular anatomies and voxel intensities; (2) severe class imbalance between foreground and background voxels; (3) image noise with different magnitudes. Limited accuracy was achieved without considering these challenges in deep learning-based methods for cerebrovascular segmentation. To overcome the limitations, we propose an end-to-end adversarial model called FiboNet-VANGAN. Specifically, our contributions can be summarized as follows: (1) to relieve the first problem mentioned above, a discriminator is proposed to regularize for voxel-wise distribution consistency between the segmentation results and the ground truth; (2) to mitigate the problem of class imbalance, we propose to use the addition of cross-entropy and Dice coefficient as the loss function of the generator. Focal loss is utilized as the loss function of the discriminator; (3) a new feature connection is proposed, based on which a generator called FiboNet is built. By incorporating Dice coefficient in the training of FiboNet, noise robustness can be improved by a large margin. We evaluate our method on a healthy magnetic resonance angiography (MRA) dataset to validate its effectiveness. A brain atrophy MRA dataset is also collected to test the performance of each method on abnormal cases. Results show that the three problems in cerebrovascular segmentation mentioned above can be alleviated and high segmentation accuracy can be achieved on both datasets using our method.

## 1. Introduction

Cerebrovascular diseases, such as strokes and aneurysms, are among the most important public health problem around the world. Although prevalence of lethal vascular diseases such as aneurysm is relatively low, estimated to be between 1 and 5% (Brisman et al., 2006), these diseases usually have a high fatality rate (McKinney et al., 2008). In order to find suitable predictors of risk of vascular diseases, computational modeling is increasingly used. Most notably, shape characterization and analysis of hemodynamic features of vessels are becoming increasingly important in prediction of aneurysm and stenosis (Raghavan et al., 2005; Millán et al., 2007). These results are strongly determined by the modeled geometry of vessels. Therefore, accurate vascular segmentation is of vital importance.

Traditional machine learning-based methods (Hassouna et al., 2006; Soares et al., 2006; Oliveira et al., 2011; Mapayi et al., 2015; Goceri et al., 2017) include unsupervised learning methods and supervised learning methods. In unsupervised learning methods, feature models for target should be proposed to distinguish vessels from background. While in supervised learning methods, pixel-wise classification in training and testing is very time consuming. Also, feature engineering is unavoidable, which is tedious and requires expertise of target domain. Recent advances in deep learning have enabled training of complex methods including deep convolutional neural networks (CNNs). Various CNNs have been proposed (Lee et al., 2015; Xie and Tu, 2015; Dou et al., 2016; Milletari et al., 2016; Christ et al., 2017; Shelhamer et al., 2017; Yu et al., 2017) in medical image segmentation. These methods require no hand-tuned image features and can be plug and play on different dataset. However, different characteristics usually exist in different dataset. If these dataset-related problems are considered in the designing and training of CNNs, higher segmentation accuracy can be realized.

Actually, in medical image segmentation, three main challenges exist. First, large variations of anatomies (Nain et al., 2004; Zheng et al., 2012, 2014; Lugauer et al., 2014; Zheng, 2016) and voxel intensities exist (Iqbal, 2013). For cerebrovascular segmentation using MRA images, large variation of vascular radius exists among different vascular branches (Rätsep et al., 2016). This variation usually corresponds to the large variation of blood flow rate among vessels (Zarrinkoob et al., 2015). Due to the imaging principle of MRA where higher blood flow rate will result in higher voxel intensity, large variations of voxel intensities are also obvious among vessels. Second, severe class imbalance makes the learning-based methods converge to biased local minima (Milletari et al., 2016; Buda et al., 2018), leading to compromised performance. In our dataset, less than 0.3% voxels belong to vascular regions. Third, image noise is an unavoidable phenomenon (Li et al., 2017), which deteriorates the quality of images and further suppresses the segmentation performance of various methods.

In this paper, we take the above problems of cerebrovascular segmentation into consideration for network designing and training. A model called voxel-wise adversarial FiboNet, termed as FiboNet-VANGAN, is tailored to relieve these problems. Specifically, our contributions are summarized as follows:

- Adversarial training is incorporated and a voxel-wise adversarial network is proposed to relieve the problem of large variations in vascular anatomies and voxel intensities.
- In order to relieve the problem of class imbalance, we propose to use the addition of cross-entropy and Dice coefficient (DC) as the loss function of the generator. Moreover, focal loss is employed as the loss function (FL) of discriminator to relieve the problem of class imbalance.
- We propose a new feature aggregation-based generator called FiboNet. By applying DC as one of the loss functions of FiboNet, the noise robustness of FiboNet is improved.

Experimental results validate the effectiveness of our contributions and segmentation results with high accuracy are achieved by our proposed model on the Healthy Dataset and the Brain Atrophy Dataset.

## 2. Related Works

Popular networks, such as U-Net (Ronneberger et al., 2015) and V-Net (Milletari et al., 2016) can be adapted to carry out cerebrovascular segmentation. However, these networks were initially proposed to deal with organ segmentation. In organ segmentation, target organs usually tightly distribute in an image volume, which is quite different from the cerebrovascular segmentation. The backbones of these two networks incorporate many max-pooling layers (four max-pooling layers are adopted in both networks) on the encoding path. Hence, networks can encode more multi-scale informative features. On the decoding path, corresponding de-convolutional layers are included to decode these features back to the scale of input image. Both of these networks use image of the full size (or cropped sub-image) as the inputs to train the networks. However, in cerebrovascular segmentation, the size of each MRA volume is much larger than the dataset of U-Net and V-Net, making it improper to apply the image of full size as the input to train networks due to limited graphic memory. Also, unlike the distribution of organs, vessels are thin and distribute sparsely and extensively within the brain. The smallest sub-image we can extract is the bounding box of the brain, which is still quite large. Therefore, to apply these two networks on cerebrovascular segmentation, patch-wise training strategy is adopted. Actually, in cerebrovascular segmentation, patch-wise training strategy may be regarded as an implicit method of data augmentation. From a global point of view, the whole cerebral vasculatures are quite different with each other intersubjectively. It is difficult for networks to learn the distribution of the whole cerebral vasculature using limited number of data. But when it comes to a local point of view, partial cerebral vasculatures are likely to share similar interhemispheric and intersubjective features, which makes it very suitable to apply patch-wise training strategy.

Aside from these two networks, several CNN-based methods have been proposed for vascular segmentation (Merkow et al., 2016; Chen et al., 2017a; Yu et al., 2017; Tetteh et al., 2018). In Merkow et al. (2016), they derived their 3D vascular network from a multi-scale 2D network for detection of boundaries. The technique called deep supervision (Lee et al., 2015) is combined and is placed at each scale to improve the performance of their network. However, they proposed no methods to deal with any of the challenges mentioned above. Hence, only limited results can be achieved. In Yu et al. (2017) adopted dense connection (Huang et al., 2017) as the backbone of their network. This type of feature aggregation is deemed to promote the training of networks. Hence, compared with networks without dense connection, their network can achieve better segmentation results. In Tetteh et al. (2018) discussed the problem of class imbalance in this application. A class balancing loss function was employed to train their network. But they did not deal with the other two problems. Chen et al. (2017a) proposed to use convolutional autoencoder to reduce the influence of noise. But they mentioned no methods to mitigate the problem of class imbalance. None of the above networks took the large variations of vascular anatomies and voxel intensities into consideration.

Actually, the problem of anatomical variation also occurs in other vascular systems (Nain et al., 2004; Zheng et al., 2012, 2014; Lugauer et al., 2014; Zheng, 2016). Data-driven approaches (Lesage et al., 2009; Schaap et al., 2009) are capable of addressing the problem of anatomical variation due to their intrinsic bottom-up paradigm. But this type of algorithm may terminate early since no or little high-level prior information is used (Zheng et al., 2013). In Zheng et al. (2014), a part based model is proposed. The anatomical variations of the pulmonary vein (PV), e.g., the left common PVs vs. separate PVs, can be addressed using this model. Similar model-driven methods are also applied to address the anatomical variations in segmenting coronary arteries (Nain et al., 2004; Zheng et al., 2012; Lugauer et al., 2014). Zheng (2016) also proposed a hybrid method in segmenting coronary arteries. Model-based approach exploiting shape priors is utilized to extract the major coronary arteries, followed by a data-driven approach to address the anatomical variations in side branches.

## 3. Methodology

In section A, we present our novel CNN-based generator (*G*) called FiboNet. The derivation of Fibonacci connection will be described. In section B, we propose an adversarial network as the discriminator (*D*). *D* is adopted as the regularizer for voxel-wise distribution consistency between the predictions from FiboNet and the ground truth. Training procedure and loss functions will be detailed in section C.

### 3.1. FiboNet as Generator

**Figure 1:**
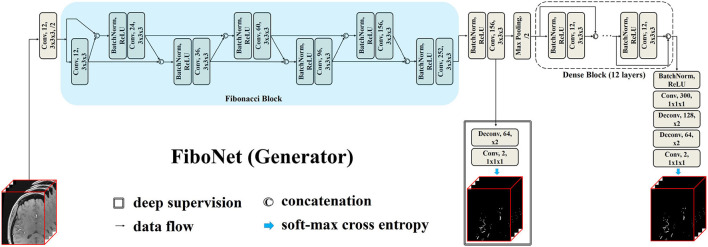
Architecture of our proposed FiboNet as the generator. Fibonacci connection of feature maps are utilized to build the Fibonacci block. In Fibonacci block, feature maps from previous two layers are concatenated as the input of the current layer. The technique called deep supervision, which is commonly used in convolutional neural network (CNN)-based medical image segmentation, is also incorporated.

The proposed FiboNet is illustrated in Figure 1. In CNNs, shallow layers are responsible for extracting low level features, such as edges and curves of different orientations and sizes, while deep layers are responsible for generating semantic information (Krizhevsky et al., 2012). If low level features are similar with each other, only limited kinds of semantic information can be generated by deep layers. Therefore, if a convolutional block can learn to extract diversified feature maps from a certain input image, very different high-level features can be represented. In the problem of cerebrovascular segmentation, different high-level features can help relieve the problem of large variation of vascular anatomies and voxel intensities.

#### 3.1.1. Fibonacci Connection and Fibonacci Block

Fibonacci connection is derived from the dense connection (Huang et al., 2017). Dense connection can be formulated as:


(1) $$x_{l}=H_{l}([x_{l-1},x_{l-2},...,x_{0}])$$

where *x*_*i*_ represents feature maps generated by the *i*^*th*^ layer. *H*_*l*_(·) represents a composite function from *l*^*th*^ layer, including batch normalization (Ioffe and Szegedy, 2015), rectified linear unit (Nair and Hinton, 2010), and convolution. By this formulation, the constraints from the current layer will directly influence each of its previous layers. Similar feature maps will be generated by each convolution layer within the same dense block (Chen et al., 2017b) due to its strong regularizing effects (Dolz et al., 2018). This may result in similar feature maps shared across different layers within the same dense block. In cerebrovascular segmentation, large variations of vascular anatomies and voxel intensities exist. Hence, we are desiring for diversified features from a certain input image. We achieve this goal by weakening the strong regularizing effect of the dense connection. We cut off some of the feature connections so that constraints from the current layer cannot directly influence each of its previous layers. Specifically, in our case, we concatenate the feature maps from previous two layers only, which is formulated as:


(2) $$x_{l}=H_{l}([x_{l-1},x_{l-2}])$$

In this type of feature aggregation, only the previous two layers are directly affected by current layer. This type of feature aggregation coincides with the developing manner of Fibonacci numbers. Therefore, we term this type of feature aggregation as Fibonacci connection. In Fibonacci connection, layers before *l* − 1, *l* − 2 are indirectly influenced. The regularizing effect of the dense block is weakened. Since diversified feature maps are extracted by this type of feature connection, it can help detect more kinds of vascular voxels.

#### 3.1.2. FiboNet

Using Fibonacci connection, we propose the FiboNet, as illustrated in Figure 1. In order to detect the thin boundaries of vessels, small kernel size is used in the whole network. In shallow layers of the FiboNet, we are expecting for weak regularizing effect between different layers in order to generate diversified feature maps and mitigate the under-detection of candidate vascular voxels.

Hence, we use the Fibonacci block in shallow layers. While dense block is placed in deep layers to extract similar high-level features belonging to vascular voxels and to avoid as much over-detection of background voxels as possible. Notably, we included a branch at the output of FiboNet as deep supervision (Lee et al., 2015), allowing gradients to inject directly into the preceding layers to better train the network (Lee et al., 2015).

### 3.2. Voxel-Wise Adversarial Network as Discriminator

In cerebrovascular segmentation, large variations exist in vascular anatomies and voxel intensities within even the same subject. In MRA images, signals with high intensity spread in the vessels with large radius. Therefore, signals with low intensity are comparatively harder to be segmented because signals with lower intensity are contained within smaller vessels, which consist of fewer voxels. Even though patch-wise training strategy is adopted, the influence of these voxels is easy to get overwhelmed by background voxels. Since a thorough knowledge of anatomy is required to understand and analyze the cerebrovascular system (Nowinski et al., 2009) with respect to different clinical purposes, such as automatic diagnosis and surgical planning, it is important to improve the segmentation performance of networks within these regions.

Recent development of deep learning has validated the effectiveness of GAN (Goodfellow et al., 2014; Luc et al., 2016) in regularizing higher order consistency (Luc et al., 2016) including the category consistency between generated images and images from the training dataset (Goodfellow et al., 2014) and the texture consistency between generated images and real images (Isola et al., 2017). GAN can be regarded as a training framework. In this framework, the generator (*G*) and the discriminator (*D*) are jointly trained. This training procedure will make the distribution generated by *G* close to the real data distribution. In Goodfellow et al. (2014), whole image is input to the *D* and *D* outputs one digit to represent the source of its input. This type of adversarial training was usually applied in regularizing category consistency. Isola et al. (2017) proposed another adversarial strategy by constraining the receptive field of each output voxel of *D* to a patch of *D*′*s* input. By this operation, they can impose texture consistency between generated images and real images. In our case, we embrace the similar idea and propose a voxel-wise adversarial network, termed as voxel-wise adversarial network (VAN), to incorporate voxel-wise distribution consistency by constraining the receptive field of each output voxel of *D* to a voxel of *D*′*s* input. Using this type of adversarial training, the segmentation performance of networks in small vessel regions can be consistently improved.

**Figure 2:**
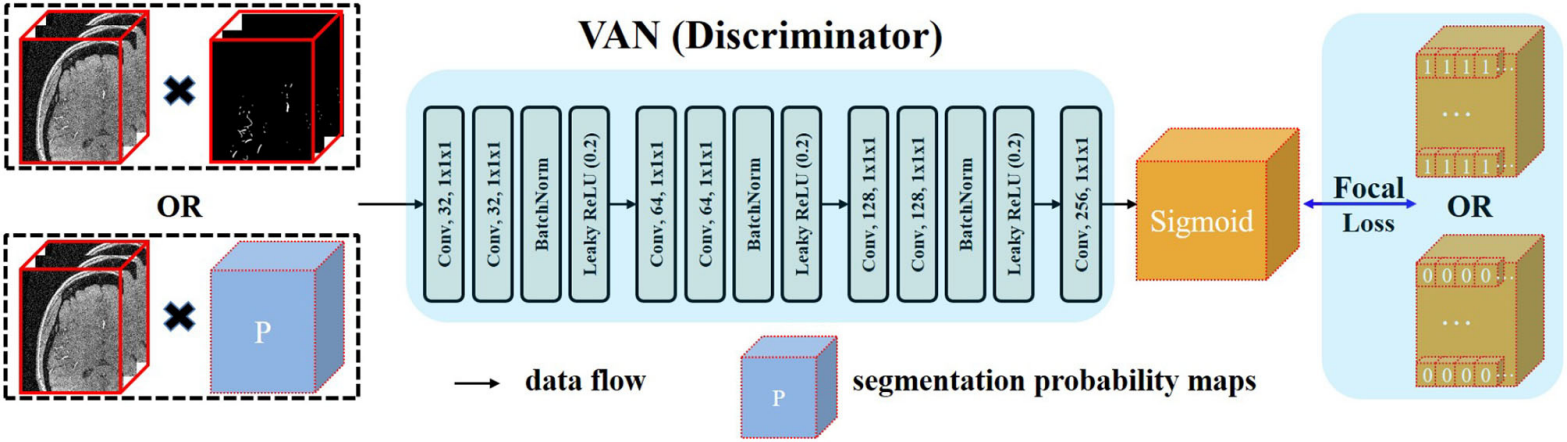
Architecture of the proposed voxel-wise adversarial network (VAN). VAN is trained to distinguish the source of its input. In the context of generative adversarial network (GAN), the procedure of adversarial training is to minimize the discrepancy between two distributions. Since we want voxel-wise similar distribution between the distribution generated by FiboNet and the real data distribution, we input these two types to VAN. Kernel size of 1 is used in VAN. FL is utilized as the loss function to relieve the problem of class imbalance.

Details of the architecture of VAN are illustrated in Figure 2. Our VAN accepts two types of inputs: one is the multiplication of noisy raw patches and the segmentation probability maps; the other is the multiplication of noisy raw patches and the annotated ground truth patches. Noisy raw patches are used as part of *D*′*s* input in case of the degenerate distributions generated by *G* (Sønderby et al., 2016). Multiplication operation is used so that VAN can take the relationships of raw patches and the predictions/ground truth into consideration from the beginning. Thus, all the model parameters are jointly trained using information from both the segmentation and raw patches.

### 3.3. Training and Losses

**Figure 3:**
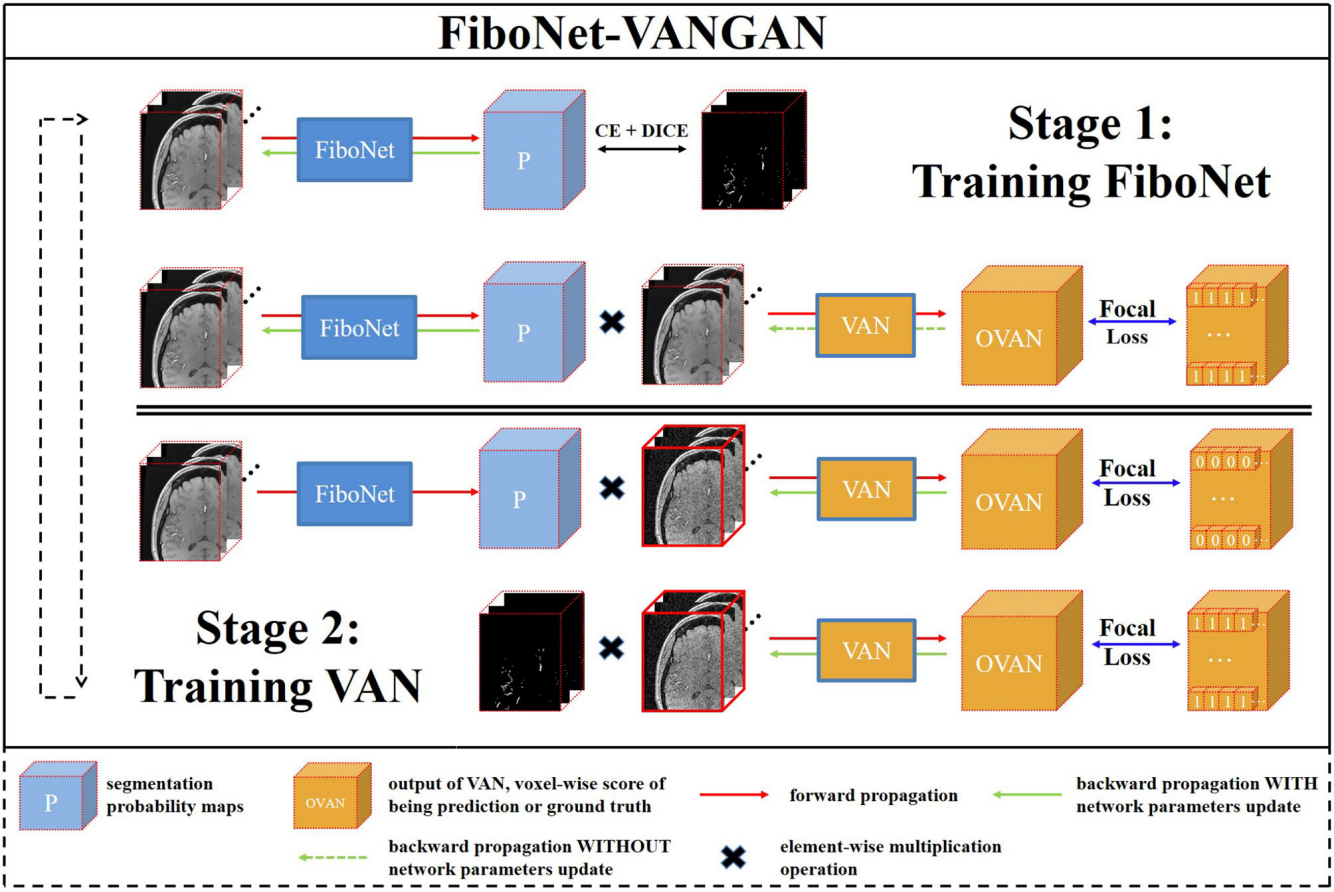
An overview of our proposed voxel-wise focal generative adversarial network (GAN) for cerebrovascular segmentation. Two stages are included in one training iteration. FiboNet is trained in stage one and voxel-wise adversarial network (VAN) is trained in the stage two.

The training pipeline of the proposed FiboNet-VANGAN is illustrated in Figure 3. The following two stages are repeated to iteratively train the generator and discriminator.

#### 3.3.1. First Stage of Training

In this stage, *G* is trained to generate segmentation probability maps close to the ground truth and also is encouraged to fool *D* about the source of its input. The loss function *L*_*G*_ is formulated as:


(3) $$\begin{array}{l} L_{G}=\sum_{i=1}^{N}l_{CE}(P_{i},Y_{i})-l_{DC}(P_{i},Y_{i})-\\ \;\sum_{i=1}^{N}l_{FL}(S_{i}=D(X_{i}\cdot P_{i}),T_{i}=0) \end{array}$$

where *N* represents the number of input patches, *P*_*i*_ represents the generated probability map of *G*′*s* softmax layer, and *Y*_*i*_ represents the corresponding ground truth. *S*_*i*_ represents the predicted score maps of *D*, and *T*_*i*_ represents target score maps of *D*. *CE* represents cross-entropy, and *DC* represents Dice

Coefficient *FL* represents focal loss and these three losses can be formulated as:


(4) $$l_{CE}(P_{i},Y_{i})=-\sum_{c=0}^{1}\sum_{v}^{V}y_{c,v}log(p_{c,v})$$

(5) $$l_{DC}(P_{i},Y_{i})=\sum_{c=0}^{1}\frac{2\sum_{v}^{V}p_{c,v}y_{c,v}}{\sum_{v}^{V}p_{c,v}^{2}+\sum_{v}^{V}y_{c,v}^{2}}$$

(6) $$l_{FL}(S_{i},T_{i})={\left(1-S_{i}\right)}^{\gamma }\cdot l_{CE}(S_{i},T_{i})$$

where *V* represents the number of voxels in current patch *P*_*i*_, *y*_*c,v*_ represents target probability of *v*^*th*^ voxel belonging to *c*^*th*^ class, and *p*_*c,v*_ represents generated probability of *v*^*th*^ voxel belonging to *c*^*th*^ class. In practice, one-hot encoding is adopted. Hence, *y*_*c,v*_ = 1 is used. γ is a decaying factor and γ = 4 is used in our implementation.

*CE* enforces voxel-wise similarity between predicted distribution of *G* and real data distribution. FiboNet trained with only *CE* tends to get trapped in biased local minima (Milletari et al., 2016) with the existence of severe class imbalance, because voxel-wise penalization is very sensitive to class imbalance. To handle this, we propose to use the addition of *CE* and *DC* as the loss function. As *DC* will incur penalization on overlapping areas, which can be concluded from Equation (5), the problem of class imbalance could be alleviated. When predictions completely overlap with ground truth, *DC* will output 1. When there are no intersections between predictions and ground truths, *DC* will output 0. In practice, we minimize (1 − *DC*).

*FL* is a weighted version of traditional binary cross entropy. In our case, voxel-wise adversarial training is employed to model steep regions in which *D* is trained to distinguish the source of its input voxel-wisely. Therefore, the problem of class imbalance will also have an influence on the training of *D*. Thus, we should take this problem into consideration in the adversarial training procedure of both *G* and *D*. During the training of *D*, it is desired that major components of loss values comes from those falsely distinguished voxels. Due to the severe class imbalance, *D* will learn quickly to distinguish the source of background voxels. Under this circumstance, loss values from foreground voxels are desired to be the major component of the total loss. But the number of these voxels are too small. Their total loss is overwhelmed by the total loss from the background voxels though these background are truely distinguished. Therefore, we need to lower the contributions of those correctly distinguished voxels. In Lin et al. (2017), the FL is proposed to improve the detection accuracy of single-stage detectors. Inspired by their work, we apply FL as the loss function of our VAN to relieve the problem caused by class imbalance. With this loss function, when *D* can output scores close to target scores of each background voxel, the weighted factor will decrease their contributions to the total loss. Thus, loss values from falsely distinguished voxels will be the major component.

#### 3.3.2. Second Stage of Training

In this training stage, *D* is trained to distinguish the sources of its input voxel-wisely. Two types of inputs are accepted. One is the multiplication of the segmentation probability maps and noisy raw patch. The other is the multiplication of the ground truth and raw patch added with noise. When the first one is input into *D*, it is desired to be recognized as 0 voxel-wisely by *D*. When the second one is input into *D*, it is desired to be recognized as 1 voxel-wisely by *D*. At this training stage, we use the FL (Lin et al., 2017) as the loss function, which is formulated as:


(7) $$\begin{array}{l} L_{D}=\sum_{i=1}^{N}l_{FL}(S_{i}=D({\hat{X}}_{i}\cdot P_{i}),T_{i}=0)+\\ \;\sum_{i=1}^{N}l_{FL}(S_{i}=D({\hat{X}}_{i}\cdot Y_{i}),T_{i}=1) \end{array}$$

where $\hat{X_{i}}$ represents the noisy raw patch. Noisy patch is used as part of *D*′*s* input in case of degenerate distribution generated by *G* (Sønderby et al., 2016). In the beginning of training *G*, a good prediction result cannot be achieved. Obvious differences exist between *P*_*i*_ and *Y*_*i*_. Therefore, the two predictions, $D\left({\hat{x}}_{i}\cdot P_{i}\right)$ and $D\left({\hat{x}}_{i}\cdot Y_{i}\right)$, of *D* are also quite different. Under this situation, the weighting factor of FL does not play a very important role. But when a large number of background voxels can be truely predicted by *G*, *P*_*i*_ and *Y*_*i*_ will be very similar. In this case, loss values calculated by *D* will be biased since we are applying adversarial training voxel-wisely. When *G* is trained with the loss value from biased *D*, the accuracy of *G* may drop. But the weighting factor in FL can make a balance between the foreground and the background voxels with the occurrence of class imbalance. It can lower the contributions of background voxels. Thus, the problem of biased convergence can be relieved by a large extent.

## 4. Experiments and Results

### 4.1. Datasets and Preprocessing

Two datasets, acquired on a 1.5 T GE BRIVO MR355 using gradient echo sequence with repetition time of 26 ms and echo time of 6.8 ms, are collected and used for method evaluation in this work, the healthy dataset and the brain atrophy dataset. Procedures of data collections were reviewed by Datian County Hospital Ethics Committee. Participants provided informed consent to participate in the study. Two clinicians were responsible for the annotation of ground truths. A consensus between them was used as the final reference standard.

#### 4.1.1. Healthy Dataset

This dataset contains 12 healthy time-of-flight MRA (TOF-MRA) volumes. Each volume is reconstructed into a matrix of size 1, 024 × 1, 024 × 92 with voxel size of 0.264 × 0.264 × 0.8*mm*. No contrast agent is used during the scanning. No removal of bias field is carried out. The age and gender distribution of this dataset are listed in Table 1.

**Table 1:** The age and gender distribution of the healthy dataset.

**Healthy dataset**				
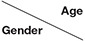	**(20,30]**	**(30, 40]**	**(40, 50]**	**Total**
Male	3	1	1	5
Female	1	3	3	7
Total	4	4	4	12

#### 4.1.2. Brain Atrophy Dataset

This dataset was collected to test the performance of each network on abnormal dataset, contains 9 TOF-MRA volumes from 9 subjects diagnosed with brain atrophy. Each volume within this dataset is reconstructed into a matrix of 512 × 512 × 128 with voxel size of 0.43 × 0.43 × 0.7*mm*. The age and gender information of this group is listed in Table 2. Atrophy of any tissue within the brain means a decrement in the size of its cells. Atrophy can be generalized, which means that all of the brain has shrunk; or it can be focal, affecting only a limited area of the brain and resulting in a decrease of the functions that area of the brain controls. In either case, the distribution of the cerebral vasculature becomes quite different from the healthy subjects even within the same range of ages. Therefore, this dataset makes a good testbed for the performance of each network on abnormal dataset.

**Table 2:** The age and gender distribution of the brain atrophy dataset.

**Brain atrophy dataset**			
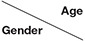	**(70,80]**	**(80, 90]**	**Total**
Male	4	0	4
Female	2	3	5
Total	6	3	9

#### 4.1.3. Data Normalization

Before training and testing, we carry out the procedure of data normalization using the following equation:


(8) $$X=\frac{X-mean(X)}{std(X)}$$

where *X* represents each MRA volume.

#### 4.1.4. Data Splitting and Patch-Wise Training/Testing

The MRA volumes are too large to fit in our graphic memory during training and testing. Also, cerebral vessels usually share similar interhemispheric and intersubjective features. Therefore, we turn to patch-wise training strategy, which has been frequently used in the context of medical image segmentation (Merkow et al., 2016; Yu et al., 2017; Gibson et al., 2018; Han et al., 2018; Kushibar et al., 2018; Tetteh et al., 2018; Wang et al., 2018). Each volume from the healthy dataset is split into overlapping patches with size of 64 × 64 × 64. An overlapping size of 4 × 4 × 36 is adopted, thus corresponding to 17, 17, and 2 patches in each direction and 578 patches in total. For each volume from the brain atrophy dataset, the same patch size is used while overlapping sizes are changed to 4 × 4 × 32 to avoid zero-padding in the axial direction, thus corresponding to 9, 9, and 3 patches in each direction and 243 patches for each MRA volume.

In the training stage, each MRA volume is split into patches to train the networks. In the testing stage, each volume is first split into corresponding patches. Then the patches are input to the networks. After the processing of networks, predicted patches are stitched into the dimension of the original testing volume. Major voting strategy is utilized to determine the categories of the voxels from overlapping regions.

All the networks are trained on the same 6 volumes, which are randomly selected from the healthy dataset. The rest 6 volumes of the healthy dataset are used as the testing dataset to test the segmentation performance of each network. The whole brain atrophy.

Dataset is utilized to evaluate the performance of each method on abnormal dataset. Thus, each network is trained on the same 6 volumes and tested on the same 15 volumes.

### 4.2. Experiment Setup

We compare our results with popular networks in 3D medical image segmentation including 3D-UNet, a 3D version of U-Net (Ronneberger et al., 2015), V-Net (Milletari et al., 2016), I2I-3D (Merkow et al., 2016), UCeption (Sanchesa et al., 2019), DenseVoxNet (Yu et al., 2017), and DeepVesselNet (Tetteh et al., 2018). We implement the derived 3D version of U-Net, and UCeption in tensorflow. As for other networks, original open source codes are used in the experiments.

In order to validate our proposal of using the addition of cross-entropy and DC, we also train both the DenseVoxNet and the FiboNet using addition of cross-entropy and DC as loss function (DenseVoxNet-CE-DC and FiboNet-CE-DC in Table 3).

**Table 3:** Quantitative comparisons of different methods on different datasets.

**Methods**	**Healthy dataset**			**Brain atrophy dataset**		
	***DC***	***SASD(mm)***	***SHD95(mm)***	***DC***	***SASD(mm)***	***SHD95(mm)***
3D-UNet Ronneberger et al., 2015	0.7133 ± 0.0349	1.0447 ± 0.2428	7.4281 ± 1.3958	0.6478 ± 0.0264	1.5247 ± 0.4347	6.8728 ± 1.7996
V-Net Milletari et al., 2016	0.7255 ± 0.0320	0.7974 ± 0.1716	4.4059 ± 1.5024	0.5842 ± 0.0307	1.1460 ± 0.3138	6.8811 ± 2.1470
I2I-3D Merkow et al., 2016	0.7298 ± 0.0269	0.9267 ± 0.2282	6.3634 ± 1.8695	0.5979 ± 0.0321	1.3444 ± 0.5033	6.6167 ± 2.8714
DeepVesselNet Tetteh et al., 2018	0.7391 ± 0.0261	1.0805 ± 0.3400	6.6839 ± 1.1783	0.6188 ± 0.0282	1.6308 ± 0.5314	8.7423 ± 2.7256
UCeption Sanchesa et al., 2019	0.7680 ± 0.0250	0.7239 ± 0.1472	4.8261 ± 1.2030	0.6036 ± 0.0198	1.9772 ± 0.3509	12.2734 ± 1.3957
DenseVoxNet-CE Yu et al., 2017	0.7611 ± 0.0227	0.6523 ± 0.1296	4.1740 ± 1.4962	0.6524 ± 0.0086	1.4203 ± 0.2831	9.7092 ± 1.9996
DenseVoxNet-CE-DC	0.7840 ± 0.0266	0.8249 ± 0.2071	5.5383 ± 1.3207	0.7120 ± 0.0224	1.3878 ± 0.3092	9.5628 ± 1.9789
FiboNet-CE	0.7985 ± 0.0216	0.4699 ± 0.1110	2.7348 ± 1.2129	0.6985 ± 0.0110	1.0479 ± 0.2122	6.5655 ± 2.4639
FiboNet-CE-DC	0.8093 ± 0.0296	0.4848 ± 0.1447	2.9483 ± 1.2064	0.7532 ± 0.0190	1.3338 ± 0.2442	9.0029 ± 1.7674
LinearCN-CE-DC	0.8016 ± 0.0264	0.5032 ± 0.1098	3.0064 ± 1.0408	0.7292 ± 0.0194	1.1558 ± 0.2092	7.4721 ± 2.1517
FiboNet-PatchGAN	0.8188 ± 0.0269	0.4247 ± 0.1073	2.6115 ± 0.9309	0.7519 ± 0.0193	1.1599 ± 0.2472	7.9665 ± 2.2705
FiboNet-VolumeGAN	0.8153 ± 0.0278	0.4486 ± 0.1272	2.6436 ± 1.0850	0.7515 ± 0.0227	1.2192 ± 0.2588	8.2716 ± 2.1943
**FiboNet-VANGAN**	**0.8197 ± 0.0300**	**0.4019 ± 0.1111**	**2.4391 ± 0.9615**	**0.7571 ± 0.0173**	**1.0023 ± 0.2508**	**6.3991 ± 2.6469**

*For the healthy dataset, results based on the rest 6 MRA volumes for testing are summarized. Bold values indicate best segmentation performance*.

In order to compare our Fibonacci connection with traditional linear convolutional (LinearCN) block where each layer only connects to its previous one, we also construct a LinearCN block by connecting each layer in Fibonacci block to its very preceding layer only.

VoxelGAN (the proposed VAN), PatchGAN, and VolumeGAN are three types of discriminators (Isola et al., 2017). In order to get a better understanding of the different influences from different adversarial training, we also carry out the experiments of different adversarial training by changing the proposed VAN to corresponding adversarial networks. Essentially, they are all fully convolutional networks but differ in the receptive field of the output voxel. When the receptive field corresponds to a voxel, it is termed as VoxelGAN. When the receptive field corresponds to a patch, it is termed as PatchGAN. When the receptive field corresponds to a volume, it is termed as VolumeGAN. In designing the architectures of PatchGAN and VolumeGAN, all convolutions are of kernel size 3 (or larger) with stride 2 (or larger) to expand the receptive field of voxels from follow-up layers. But in designing the architecture of VoxelGAN, kernel size 1 and stride 1 are adopted to restrict the receptive field. In our experiments, architectures of PatchGAN and VolumeGAN similar to Isola et al. (2017) are adopted:

- PatchGAN: *C*64 − *C*128 − *C*256
- VolumeGAN: *C*64 − *C*128 − *C*256 − *C*512 − *C*512

where *Ck* denotes a Convolution-BatchNorm-ReLU with *k* filters. All ReLUs are leaky with slope 0.2. The receptive field of a output voxel in PatchGAN is 15 × 15 × 15 and VolumeGAN 63 × 63 × 63.

All experiments were performed on workstation, which runs Ubuntu 16.04 operation system and is equipped with an Intel Core i7-5960 CPU (3.50 GHz), 32GB RAM, and a NVIDIA GeForce 1080 Ti video card with 11GB graphics memory. The SGD solver with learning rate 0.001, which exponentially decays by 10% every 5 epochs, and momentum parameters β_1_ = 0.9, β_2_ = 0.999 are used for training all the adversarial networks. Each network is trained for 40 epochs to ensure convergence.

To quantitatively evaluate the performance of each method, three metrics are adopted to evaluate the segmentation accuracy:

- Dice coefficient (*DC*) Dice (1945):
  *DC* = 2|*P* ∩ *G*|/(|*P*| + |*G*|)
- Symmetric Average Surface Distance (*SASD*) Yeghiazaryan and Voiculescu (2015):
  *SASD* = (*mean*(*Dist*(*P, G*)) + *mean*(*Dist*(*G, P*)))/2
- Symmetric 95% Hausdorff Distance (*SHD*95) Dubuisson and Jain (1994):
  *SHD*95 = (*P*_95_(*Dist*(*P, G*)) + *P*_95_(*Dist*(*G, P*)))/2

where *P* represents the predicted segmentation maps of each network, *G* represents the annotated ground truth, *Dist*(*P, G*) is the set of distances from boundary voxels of *P*, δ_*P*_, to the nearest boundary voxel in δ_*G*_, i.e.,:


(9) $$Dist(P,G)=\{\underset{x\in \delta_{P}}{\min}\|x-y{\|}^{2}|y\in \delta_{G}\}$$

wherein *P*_95_(*Dist*(·)) is the 95th percentile of *Dist*(·).

Among these metrics, the *DC* is a commonly used metric for measuring the general agreement between the generated segmentation maps and the annotated ground truth. It can be utilized to evaluate the general performance of each method. *SASD* reflects the globalized average boundary agreement, while *SHD*95 is usually applied to evaluate the localized disagreement.

### 4.3. Qualitative Results

**Figure 4:**
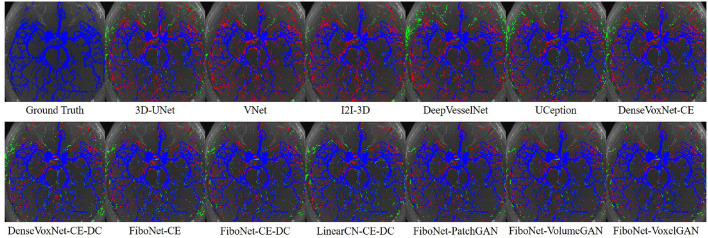
Maximum intensity projections of each method. Pixels in blue represent true positives. Pixels in green represent false positives. Pixels in red represent false negatives.

**Figure 5:**
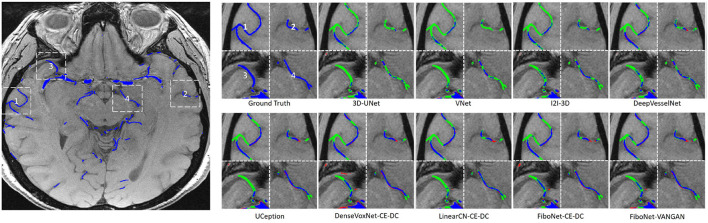
Visual results of the four different white dash-line rectangles of each method. Pixels in blue represent true positives. Pixels in green represent false positives. Pixels in red represent false negatives.

Maximum intensity projection, along with slice-wise result, of each method was shown in Figures 4, 5, respectively, wherein blue, red, and green pixels represent true positives, false negatives, and false positives, respectively.

### 4.4. Quantitative Results

In Table 3, we listed the quantitative comparisons of different methods. Aside from the results on the rest 6 testing volumes from the healthy dataset, we also include the results for the 9 testing volumes from the brain atrophy dataset.

### 4.5. Statistical Analysis

Paired-sample *t*-test is adopted to evaluate the significance of the improvement of the average *DC* by the proposed method. We contrast the proposed FiboNet-VANGAN with other methods listed in Table 3. One-tailed results are reported since we only care about the significance of the improvements. Therefore, our null and alternative hypothesis should format separately as:


(10) $$H_{0}:\mu_{1}=\mu_{2}$$

(11) $$H_{\alpha =0.01}:\mu_{1}>\mu_{2}$$

where *H*_0_ represents the null hypothesis and *H*_α_ alternative hypothesis. μ_1_ represents the average *DC* value from the proposed method, FiboNet-VANGAN, and μ_2_ the contrasting method accordingly. In this hypothesis testing, we set the significance-level to 0.01. Here, we report the test results of both datasets. In the healthy dataset, μ_*i*_, (*i* = 1,2) is the average *DC* value over the 12 volumes. In the brain atrophy dataset, μ_*i*_, (*i* = 1,2) is the average *DC* value over the 9 volumes. Aside from *p*-values, we also included values of Cohen's D and intraclass correlation coefficient (ICC) to test the robustness of the proposed model. The test results are listed in Table 4, wherein *p*-values showing statistical significance, along with the corresponding values of Cohen's D and ICC, were represented by black bold fonts.

**Table 4:** *P*-value, Cohen's D, and intraclass correlation coefficient (ICC) values between the proposed method, FiboNet-VANGAN, and each of its counterparts.

***P*** **-value/Cohen's D / ICC**						
**Methods**	**Healthy dataset**			**Brain atrophy dataset**		
3D-UNet	**7.6***e*^**-7**^ / **3.26** / **0.84**			1.7*e*^**-4**^ / **4.89** / **0.93**		
V-Net	8.9*e*^**-7**^ / **3.03** / **0.82**			6.3*e*^**-7**^ / **6.94** / **0.96**		
I2I-3D	1.9*e*^**-7**^ / **3.15** / **0.83**			2.0*e*^**-6**^ / **6.17** / **0.95**		
DeepVesselNet	2.1*e*^**-7**^ / **2.86** / **0.80**			7.3*e*^**-7**^ / **5.90** / **0.95**		
Uception	1.9*e*^**-6**^ / **1.87** / **0.83**			2.6*e*^**-6**^ / **8.25** / **0.97**		
DenseVoxNet-CE	1.3*e*^**-6**^ / **2.19** / **0.70**			5.7*e*^**-7**^ / **7.66** / **0.97**		
DenseVoxNet-CE-DC	2.7*e*^**-8**^ / **1.22** / **0.42**			4.5*e*^**-7**^ / **2.25** / **0.73**		
FiboNet-CE	2.1*e*^**-4**^ / **0.81** / **0.24**			4.0*e*^**-4**^ / **4.04** / **0.90**		
FiboNet-CE-DC	1.3*e*^**-6**^ / **0.35** / **0.06**			4.2*e*^−1^ / 0.01 / 0.02		
LinearCN-CE-DC	1.3*e*^**-5**^ / **0.64** / **0.16**			6.1*e*^**-6**^ / **1.51** / **0.55**		
FiboNet-PatchGAN	2.9*e*^−1^ / 0.04 / 0.00			2.8*e*^−1^ / 0.28 / 0.04		
FiboNet-VolumeGAN	1.8*e*^**-3**^ / **0.21** / **0.01**			6.2*e*^**-2**^ / **0.28** / **0.04**		

*Bold values indicate improvements of statistical significance*.

In the healthy dataset, the proposed method significantly outperforms its counterparts, demonstrating a significant improvement of *DC* value by our method. In the brain atrophy dataset, significant improvement can also be observed except the result from FiboNet-PatchGAN. Indeed, FiboNet-PatchGAN can achieve comparable accuracy in the brain atrophy dataset with the proposed method, but it underperforms in the healthy dataset.

### 4.6. Evaluation of the Diversity of Feature Maps

The feature map generated by the Fibonacci block (or the dense block) is a matrix of shape [*H, W, D, C*], where *H, W, D, C* represents the value of height, width, depth, and channel, respectively. By calculating the standard deviation along the fourth dimension, we can get the standard deviation map, shaping like [*H, W, D*], of the channels. Based on the channel-wise standard deviation map, histograms are calculated to compare the diversity of feature maps generated by these two blocks.

**Figure 6:**
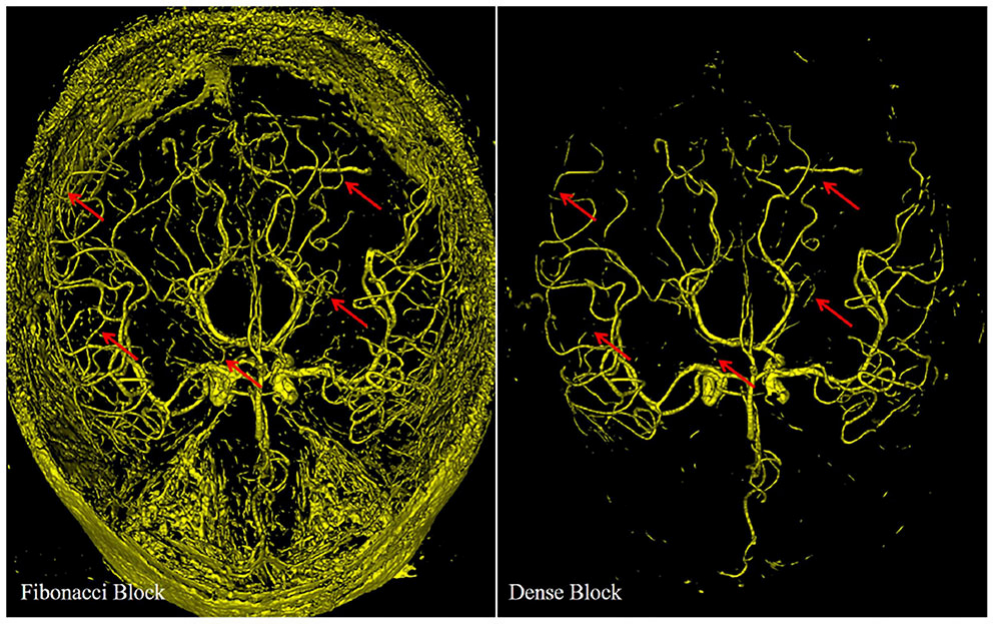
Different results from the deep supervision branch of FiboNet and DenseVoxNet, illustrating the different detection performance of Fibonacci block and the dense block. Red arrows indicate regions where FiboNet could extract more complete vasculature than DenseVoxNet.

In Figure 6, we display the output of the deep supervision branch to validate the performance of the Fibonacci block and the dense block.

Compared with the results of the dense block, more candidate voxels are extracted by the Fibonacci block. Few vascular voxels are left out by the Fibonacci block. While in the result of the dense block, though the vascular regions compose the major part of the result, distal vascular regions and many small vascular branches are missing. Actually, both results stem from minimizing the loss function. If features of distal vascular regions are learned by the dense block, these voxels will be segmented so as to reduce the loss value. But in the dense block, due to its regularizing effect, these features are probably not learned. The Fibonacci block derives from the dense block by weakening its regularizing effect, as described in section II, which leads to the improvement of the detection ability of network. This results in the segmentation of distal vascular voxels but also many background voxels. If features within these regions are not learned, there is no chance that they can be segmented. Therefore, we can conclude from this observation that diverse feature maps are generated by the Fibonacci block, that is, the FiboNet.

**Figure 7:**
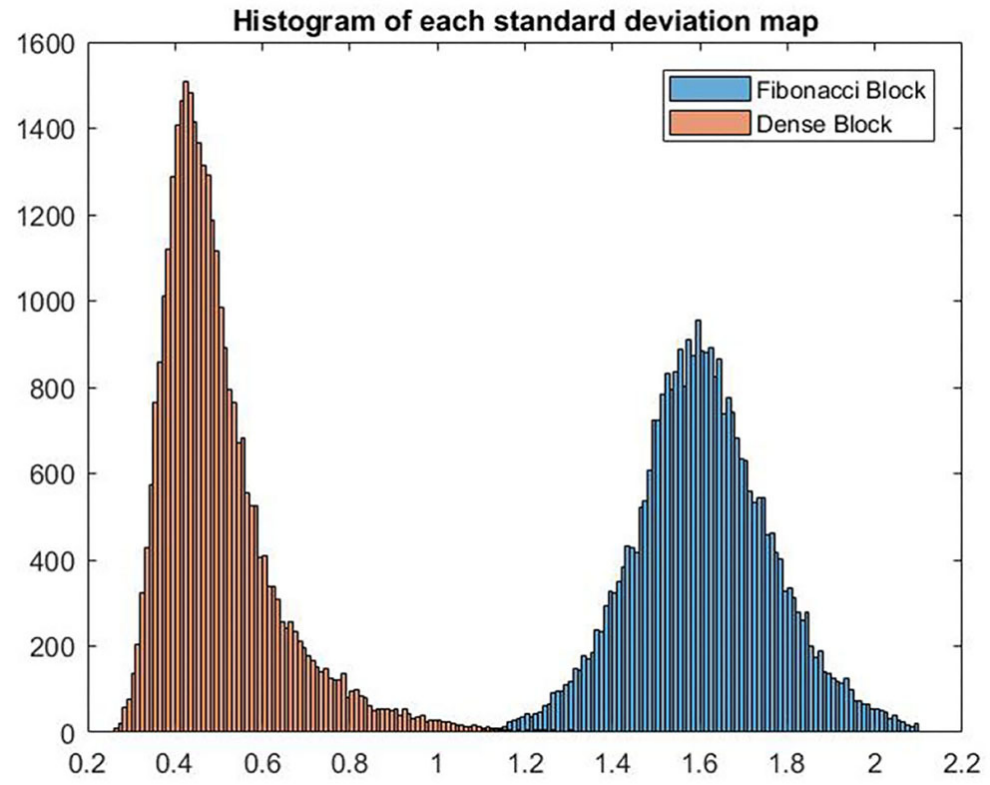
VHistograms of the two standard deviation maps calculated using the feature maps from Fibonacci block and dense block, respectively.

In Figure 7, we show the histograms of channel-wise standard deviation map calculated using the feature maps generated by Fibonacci block and dense block. It can be concluded from the histograms that standard deviations of the feature maps generated by the Fibonacci block are larger than their counterparts from the dense block, which means the Fibonacci block can generate more diversified feature maps than the dense block.

### 4.7. Noise Robustness

**Figure 8:**
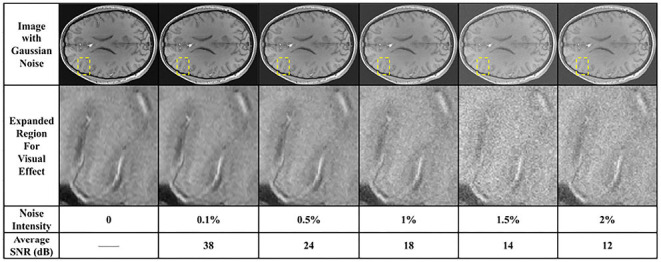
Illustration of a slice with different magnitudes of Gaussian noise. Window level is 240 and window width is 240. The region marked by yellow dash-line rectangle is expanded for better visual effects. Averaged : signal to noise ratioes (SNRs) between images with added noise and the original image were listed in the last row.

Noise is commonly observed in medical imaging. Automatic methods should be robust to different magnitudes of noise. In order to evaluate the robustness of different methods, we apply the different methods on test set with different magnitudes of noise. Gaussian noise with 0.1%, 0.5%, 1%, 1.5%, and 2% of the image magnitude is added. In Figure 8, we demonstrate a slice with no Gaussian noise and the slices with different magnitudes of Gaussian noise. A rectangle region marked by the yellow dash-line is expanded for better visual effects. The increasing magnitudes of Gaussian noise deteriorate the contrast of the original slice. Some vascular boundaries are blurred and degenerated, thus hard to be distinguished from background egions.

**Figure 9:**
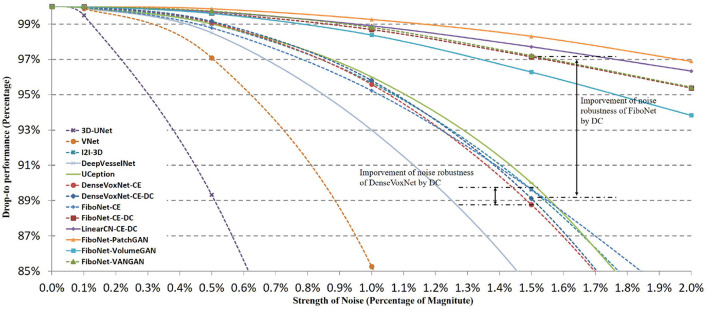
Influences of different magnitudes of Gaussian noise on the segmentation accuracy of different methods. Horizontal axis represents the strength of noise added. Vertical axis represents drop-to performance of a certain method compared with the situation when no Gaussian noise is added.

In Figure 9, we display the quantitative comparisons of noise robustness of each method. We use the vertical axis to represent the drop-to performance calculated via:


(12) $$DTP=\frac{DC_{p}}{DC_{0}}\times 100\%$$

where *DC*_*p*_ represents the *DC* value obtained from image with p% noise, and *DC*_0_ is the *DC* value when no Gaussian noise is added. The horizontal axis represents the different magnitudes of Gaussian noise added. It can be observed that the performance of each method drops with the increasing strength of noise. But the robustness of each method is quite different.

Among these networks, 3D-UNet turns out to be the least robust to noise. In this network, batch normalization is not included, which will lead to ineffective training. Dropout layer to enhance the robustness of network is not included either. An improved robustness can be observed in V-Net where residual connection of feature maps is utilized in network design. Residual connection helps promote the effective training of networks. Therefore, better robustness can be realized. However, in the implementation of V-Net, residual connection is incorporated in the first layer of each scale of feature space. No input transition layer (Smith and Topin, 2016) is used. Usually, an input transition layer is utilized for two reasons. One is to transform the input from raw image space to its corresponding feature space. The other reason is to increase the number of channels of input image, which allows the input data to be examined in many ways. This type of layer has been adopted in many successful networks (Krizhevsky et al., 2012; Szegedy et al., 2015, 2016, 2017; He et al., 2016). But in V-Net, a residual block is directly adopted after the input image. Noise from raw image is propagated to and further influence deep layers. Hence, only limited improvement is achieved. Better situations can be observed in I2I-3D, DenseVoxNet, FiboNet, and LinearCN. Within these networks, convolution layers are utilized as input transition layers to transform from raw image space to feature space. Influence of noise can be reduced by the nonlinear transformation of input transition layers. Attentions should also be paid to the improvement brought about by incorporating *DC* as one of the loss functions. In DenseVoxNet, very limited improvement of noise robustness is achieved by *DC* loss. While in FiboNet, the improvement of noise robustness is very obvious and much larger than that in DenseVoxNet. Finally, PatchGAN and VANGAN help improve the noise robustness by a small margin. Therefore, in practice we should trade off between the segmentation accuracy and noise robustness when designing network architectures.

### 4.8. Size of Training Patch

In section II, we elaborate the reason to utilize patch-wise training strategy. However, in the original implementation of U-Net (Ronneberger et al., 2015), image of size 572 × 572 is applied for training and testing. In the original implementation of V-Net (Milletari et al., 2016), image of size 128 × 128 × 64 is adopted. Due to the limited graphic memory, we cannot carry out the experiment using images of size 572 × 572 × 572 for 3D-UNet.

But we try the experiments with patches of size 128 × 128 × 64 and 64 × 64 × 64 on V-Net, LinearCN, and FiboNet. The same data splitting strategy of the healthy dataset as described in section IV.A is used. These three networks are trained for 40 epochs, which means it goes over the training dataset for 40 times either by the way when patch size of 128 × 128 × 64 is adopted or the way patch size of 64 × 64 × 64 is adopted.

The results are summarized in Table 5. It turns out that better results are obtained using patches of size 64 × 64 × 64, which in turn supports our assumption that small patches can be regarded as an implicit data augmentation for cerebrovascular segmentation due to potential similar feature from small patches.

**Table 5:** Results using different size of training patches.

**Healthy dataset**			
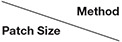	**DC**		
	**V-Net**	**LinearCN**	**FiboNet**
64 × 64 × 64	0.7191	0.7927	0.7967
128 × 128 × 64	0.6636	0.7125	0.7748
Δ*DC*	0.0555	0.0802	0.0219

## 5. Discussion

Clinically acceptable segmentation accuracy has yet to be defined for assisting surgical planning, the preventive diagnosis and quantitative analysis of cerebral vascular diseases. However, following three aspects could be the focuses for developing algorithms of cerebrovascular segmentation:

- High segmentation accuracy of vasculature—This is important for many clinical applications like invasive surgical planning, preventive diagnosis, etc. In invasive surgical planning, we should include accurate surface model to plan a proper path for intervention. In preventive diagnosis, clinicians can make a comprehensive and meaningful evaluation based on geometric features of vasculature, which should also base on high segmentation accuracy.
- Detection of vessel with small radius—Some aneurysms occur at the boundaries of small vessels. Failing to detect these vessels may lead to mis-evaluate the risk of strokes.
- Robustness to noise—Noise is an inevitable phenomenon during imaging. If automatic algorithms are not robust enough to noise, the segmentation performance will drop. This leads to inaccurate vascular information, based on which it is hard to carry out meaningful postprocessing like surgical planning, geometric analysis of vessels, etc.

In this paper, we present a deep learning-based algorithm to segment the cerebral vasculature for TOF-MRA images. Compared with the counterparts trained using CE as the only loss (DenseVoxNet-CE and FiboNet-CE in Table 3), the inclusion of *DC* improves the segmentation result by ~2%. The result of DenseNet-CE-DC is close but still lower than our FiboNet-CE, which also validates the effectiveness of FiboNet. In order to alleviate the large variations in vascular anatomies and voxel intensities, we propose a VAN. VAN is adopted as the regularizer for voxel-wise distribution consistency between the predictions and ground truth by incorporating it in the adversarial training. To mitigate the influence of class imbalance on the convergence of *D*, FL is utilized as *D*′*s* loss function. To relieve the influence of class imbalance on the convergence of *G*, the FiboNet, we propose to use the addition of cross-entropy and DC as *G*′*s* loss function. In this paper, we also propose the Fibonacci connection of feature maps and incorporate it in our FiboNet. Compared with the dense connection, this type of feature aggregation is suitable to be placed in shallow layers for diversified feature maps. Noise robustness is improved by a large margin by incorporating the DC in the training of FiboNet.

Comparatively, 3D-UNet turns out to demonstrating the weakest performance on both datasets. Maybe, only low level feature like voxel intensity is learned by 3D-UNet. From the results of V-Net and I2I-3D, conclusion can be made that, deep supervision helps improve its performance on abnormal dataset due to its intrinsic property to promote highly discriminative feature maps between convolutional layers (Lee et al., 2015), which also coincides with our motivation of Fibonacci connection. Comparatively, cross-hair filters used in DeepVesselNet helps achieve comparable segmentation accuracy but with improved memory usage and thus faster training speed.

Refer to the results of DenseVoxNet-CE-DC, FiboNet-CE-DC, and LinearCN-CE-DC for comparison of different connection types. Within these results, the DenseVoxNet-CE-DC gets the smallest *DC* value. This is probably due to the regularizing problem. The performance of LinearCN-CE-DC is a little weaker but very close to the performance of FiboNet-CE-DC. From Figure 4, we can find out that LinearCN-CE-DC performs better in vascular regions while it does worse in background regions. Worse local agreement is achieved by LinearCN-CE-DC than FiboNet-CE-DC. This can also be concluded from its value of *SHD*95, which is much larger than FiboNet-CE-DC. On the contrary, the FiboNet structure applies the feature-reusing strategy by concatenating the output from previous two layers as the input of the current layer. Compared with LinearCN, this type of connection encourages better back propagation of gradients. Compared with dense connection, this type of connection encourages diversified feature maps, which is better for vascular segmentation due to all kinds of vascular features.

The incorporation of adversarial training helps improve the segmentation performance on both datasets. The improvements from VolumeGAN is slight on both datasets. Comparatively, PatchGAN can achieve much higher improvement than VolumeGAN on the brain atrophy dataset. This may imply that small size of training patches are preferred in deep learning-based vascular segmentation since vasculatures are likely to share similar feature from a small patch instead of a large one (refer to section IV.G for details). VolumeGAN is deeper than PatchGAN, making it harder to train. Similar phenomenon that PatchGAN outperforms VolumeGAN is observed in Isola et al. (2017). Also, when networks go deeper, fewer voxels are output by VolumeGAN, making it ineffective in producing stable and sufficient gradient feedback for network training (Xue et al., 2018). The application of voxel-wise adversarial training (VANGAN) can relieve above problems. VANGAN has greater depth than VolumeGAN and the output size of VANGAN is the same as its input, ensuring sufficient backpropagation of gradient. Also, best and more consistent performance is realized on both datasets using VANGAN than that of PatchGAN. This can be concluded by the largest *DC* value and the smallest standard deviation of VANGAN among the methods.

Though improvements have been made, imperfections of our method do exist. First of all, our method only deals with the segmentation of cerebral vasculature. Centerline of the extracted vasculature cannot be detected explicitly. In applications involving the geometric information of vessels, centerlines are very important. With centerlines, the accuracy of the automatically reconstructed topology can also be measured. Second, we observe some false merges in the segmentation of vessels that are quite close to each other. When vessels are quite close to each other, only a blurry small gap of two to three voxels is supposed to separate the vessels. In the situation like this, existing methods including our FiboNet fail to separate the vessels. Instead, the blurry small gap is also recognized as vascular region, leading to false merge of vessels. Third, in patch-wise training, space information is lost for now. Networks cannot perceive the global space position of each voxel within each subject. Therefore, vessels outside the brain will also be extracted. In the future, attentions will paid to addressing above problems.

## Data Availability Statement

The raw data supporting the conclusions of this article will be made available by the authors, without undue reservation.

## Author Contributions

BG, BL, and XB conceived the project. BG implemented it and wrote the draft. BG, BL, XB, and FZ edited it. All authors contributed to the article and approved the submitted version.

## Funding

This work is supported by the National Key R&D Program of China under Grant No. 2018YFA0704101, and the National Natural Science Foundation of China (61601012, U1736217).

## Conflict of Interest

The authors declare that the research was conducted in the absence of any commercial or financial relationships that could be construed as a potential conflict of interest.

## Publisher's Note

All claims expressed in this article are solely those of the authors and do not necessarily represent those of their affiliated organizations, or those of the publisher, the editors and the reviewers. Any product that may be evaluated in this article, or claim that may be made by its manufacturer, is not guaranteed or endorsed by the publisher.
